# Report on Eye Inspection of Administrative Labor Companies

**Published:** 1919

**Authors:** 


**Report on Eye Inspection of Administrative Labor Companies.** By GEORGE S. DERBY.

In general the system of control was that adopted by the B. E. F. Trachoma was discovered among the Egyptians, Cape boys and Chinese employed by the B. E. F. There are upwards of 100,000 Chinese in France. Among this body of men 8500 cases of infectious trachoma were found, and 5500 cases of suspicious conjunctivitis.

All Chinese coming to France are routed to the Central Distributing Depot at Moyelles. They are divided into 3 classes : those with clean eyes designated as X, those with suspicious conjunctivitis designated as Y, and those with trachoma designated as Z. X, Y, and Z companies are formed and sent out. X companies may be sent anywhere in the B. E. F. Y and Z companies may be sent only to special areas where there are designated trachoma treatment centers and where these men can have the supervision and treatment of an Ophthalmic Specialist.

The technic of examination in the A. E. F. was that used by the British.

The two or three examiners stand in front of a table at which sits a clerk with a record book. On the table is water, soap and towels, so that the hands may be washed periodically. The laborers approach to be examined in single file and an inspection is made of the conjunctiva and cornea, the lids being everted in all instances and the conjunctiva and fornices inspected.

At the end of the inspection, we have a record of : (1) number examined; (2) number of cases of suspicious conjunctivitis (Y); and (3) number of cases of active trachoma (Z).

The Commander of the Company, the Medical Officer, and the Ophthalmic Specialist attached to the nearest hospital are gathered together, and the situation is thoroughly discussed and instructions given for the isolation and treatment of the Y and Z groups.

The figures show that 12461 laborers were examined and that among them were 1618 cases of conjunctivitis, 13 o/o; and 251 cases of trachoma, 2 o/o. If we separate the nationalities we find :

1. Chinese, 4958 examined; 1090 with conjunctivitis — 21 o/o; and 160 with trachoma — 3.2 o/o.

2. Indo Chinese, 1795 examined; 284 with conjunctivitis — 16 o/o; and 44 with trachoma — 2.4 o/o.

3. North Africans, 1291 examined; 51 with conjunctivitis — 4 o/o; and 18 with trachoma — 1.5 o/o.

4. Mixed, 4417 examined; 193 with conjunctivitis — 4.3 o/o; and 39 with trachoma — .9 o/o.

As a rule the trachoma seen among our Chinese was in a much earlier stage than that seen among the British. In very few cases was it disabling.

Measures should therefore be taken, first to prevent the disease from spreading, and second to prevent the trachoma which exists from developing into a type which would disable the laborer.

Trachoma cases should be isolated in special companies. These companies should be worked in some hospital area, where they can be under the supervision and care of the Ophthalmic Specialist.

Conjunctivitis cases (Y) should be isolated in special companies and given appropriate treatment. They should, as far as possible, wash separately, eat separately, and work separately from the clean men.

Local treatment consists in the administration of zinc sulphate drops (.3 o/o) twice a day.

These Y, or conjunctivitis cases, should be examined by an ophthalmic specialist every three weeks. In six weeks it should be possible to tell which of the suspicious cases are, in all probability, trachoma, and measures should be taken accordingly. Where trachoma has existed in a company, a certain number of the X or clean men will later develop it, and these must be carefully watched for.

Clean (X) Companies. The Medical Officer in charge of X companies should be on the constant lookout for fresh cases of eye disease. If these cases do not respond to treatment in a short period of time, the advice of the nearest ophthalmologist should be sought.

